# Isolation, characterization, and assessment of *Bacillus rugosus* potential as a new probiotic for aquaculture applications

**DOI:** 10.1038/s41598-024-74534-x

**Published:** 2024-10-23

**Authors:** Nermeen M. Shokrak, Nabilah Khairi, Nur Hazlin Hazrin-Chong, Radi A. Mohamed, Bahaa Abdella

**Affiliations:** 1 Faculty of Aquatic and Fisheries Sciences, Kafrelsheikh University, Kafrelsheikh, 33516 Egypt; 2 Department of Biological Sciences and Biotechnology, Faculty of Science and Technology, Universiti Kebangsaan Malaysia (UKM), Bangi, 43600 Selangor Malaysia

**Keywords:** Probiotic, *Bacillus rugosus*, Tilapia, Sustainability, Aqua feed, Gut health, Microbiology, Bacteria, Bacteriology

## Abstract

Aquaculture is an important component of the world food supply and a significant source of protein. However, this industry faces numerous problems. Including poor fish feed digestion and uneconomic nutrient utilization. This can result in unsatisfactory growth rates and poor stock performance. Utilizing probiotics, which are beneficial microbes that can enhance digestive systems and general fish health, is one possible way to address these issues. This study was designed to identify and evaluate a novel strain of *Bacillus* as a promising probiotic. The strain of *Bacillus rugosus* that was examined and coded NM007 showed promising probiotic characteristics that could help fish digest and utilize their feed more efficiently, reduce feed waste, and improve their digestive systems. *B. rugosus* NM007 exhibited the ability to produce digestive enzymes like protease, amylase, and lipase, which are the main digestive enzymes. It showed strong auto-aggregation activity and co-aggregation activity with *Aeromonas* sp. and *Streptococcus* sp. It also demonstrated tolerance to the presence of bile salt, acidic pH, and salinity up to 60 ppt. The sensitivity analysis towards antibiotics, hemolytic activity and the safety assessment on Nile tilapia fish (*Oreochromis niloticus*) confirmed the safety of this isolate. Based on the findings of this investigation and the isolate’s characterization, *Bacillus rugosus* NM007 could serve as a new promising probiotic bacterium for aquaculture.

## Introduction

Aquaculture is a crucial component of global food security, generating over 179 million tons of fish, valued at around USD 250 billion per year^1^. Notably, approximately 156 million tonnes are utilized for human consumption, providing an average of 20.5 kg of aquaculture-produced fish per person^1^. However, the sustainable growth of this industry faces multifaceted challenges, including disease outbreaks, environmental changes, and the depletion of wild fish stocks and also increasing feed costs^2–4^. The expense of fish feed constitutes a significant portion of the total expenses in aquaculture production which can reach up to 75–95% of the production cost^5^. Nevertheless, a considerable amount of this feed often goes to waste, exacerbating both economic and environmental concerns^6,7^. The inefficient digestion and utilization of feed by fish result in the release of excess nutrients into the water, contributing to pollution and potentially harming aquatic ecosystems^8^. The use of microbes that produce hydrolyzing enzymes offers a possible solution for this problem^9^. These microbes can break down complex feed components into simpler forms that are more readily absorbed by fish, thereby enhancing feed efficiency and reducing waste^10^. By implementing such microbial solutions, aquaculture operations can not only optimize their production costs but also minimize their environmental impact, promoting sustainability in the industry^11^. Addressing these challenges requires innovative approaches that integrate ecological principles with technological advancements. Among these approaches, the use of probiotics derived from enzyme-producing bacteria holds significant promise for enhancing the health and performance of aquaculture species while minimizing adverse environmental impacts^12^. In aquaculture, probiotics are defined as beneficial microorganisms, when administered through feed or aquatic environments, can benefit the host by improving the microbiome, providing disease protection, enhancing health conditions, improving growth efficiency, increasing feed consumption, strengthening stress responses, and boosting overall health^13^. Moreover, the exposure of aquatic animals to microbial cells might trigger different immunological mechanisms that help in disease tolerance^14^. Before probiotics can be effectively utilized, various characteristics need to be investigated in vitro to ensure optimal performance and potential success in real-world circumstances. These include the ability to survive in gastrointestinal conditions, adherence to the intestine, tolerance to acid and bile salts, sensitivity to antibiotics^15,16^. Moreover, the production of digestive enzymes and cell surface hydrophobicity are further criteria for evaluating probiotics in aquaculture. However, it is uncommon in nature to find all these characteristics in a single super probiotic strain, so a combination of compatible strains with varying capabilities is required. In addition to a comprehensive evaluation of the suggested strain or strains, stocking fish species and environmental factors must be taken into consideration in order to get the intended result from probiotic use. While the search for novel probiotics continues, *Bacillus* species are gaining a lot of attention in the development of aquaculture because of their advantageous qualities, which include a long shelf life, stability at high pH, resistance to UV radiation, tolerance at high and low temperatures, and well-established characteristics^17^. By isolation, identification and characterization of these bacteria for use as probiotics, researchers aim to augment the digestive efficiency, disease resistance, and overall well-being of aquaculture species, thereby promoting sustainable aquaculture practices.

In the current study a new bacterial strain was isolated and characterized for its potential application in aquaculture. The strain that quantitatively produces the key degradative enzymes was selected for further characterization. It is characterized phenotypically and genotypically, tested for probiotics successful traits and tested for its safety in vivo.

## Materials and methods

### Sample collection and processing

Fish and soil samples were collected from different locations. Soil was randomly collected below the soil surface 15 cm and kept in sterile polystyrene bags^18^. Fish samples of Nile tilapia (*Oreochromis niloticus*) and Sardine were dissected, and fish gut was collected. One gram of each sample was mixed/ homogenized with 9 mL of saline separately and then 100 µL of the homogenized samples were applied on tryptic soya broth (TSA) plates after serial dilution of the samples up to a 10^−10^. The inoculated plates were incubated at 37 ℃ for 24 h^19^. Streaking plate technique was employed for purification step. Isolated bacteria were preserved in glycerol broth at -80 ℃. The bacterial isolates were further studied based on morphological, microscopic and biochemical analysis.

### Screening extracellular enzymes production

The potential of the isolated strains for production of extracellular hydrolytic enzymes was tested as follows: The protease activity of the isolates was assessed, where tryptic soy agar (TSA) medium supplemented with 10% skimmed milk. The presence of clear zone around the colonies indicated proteolysis after 24 h of incubation at 37 ℃^20,21^. The amylase activity was assessed where TSA medium supplemented with 10% soluble starch. The presence of clear zone around the colonies indicated amylolytic activity after 24 h of incubation at 37 ℃^22^. The degradation of starch was visualized by flooding the plate with Lugol′s solution. The lipase activity was assessed according to Al-Dhabi et al. with some modification^23^, the tested strain was culture on TSA medium containing 1% soya oil. Oil degradation zone around the colonies indicated lipolysis after 24 h of incubation at 37 ℃. The degradation zone was visualized by flooding the plate with Lugol′s solution. The ratio between the average degradation zone diameter and the average colony diameter was calculated and the strains that showed the highest ratios were selected.

### Phenotypic characterization

For the selected strain, phenotypic characterization was conducted according to Bergey’s Manual, encompassing the morphology of colonies and cells, pigmentation, spore formation, response to Gram staining. Several physiological and biochemical tests were also conducted such as oxidase test, gelatin hydrolysis, methyl red and Voges–Proskauer test. In addition, the strain was tested for the ability to grow under different pH conditions and salinities.

### Molecular characterization

A single colony was inoculated in nutrient broth and grown overnight at 37 ℃ before the bacterial cells in the liquid culture were collected by centrifugation. DNA was extracted using the MagPure Bacterial DNA extraction kit (Magen, China) based on manufacturer protocol. The purity and quantity of DNA were examined by using Thermo Scientific NanoDrop One Microvolume UV-Vis spectrophotometers. The DNA was used as a template DNA for amplification of the 16 S rRNA gene. The universal primers used for DNA amplification were 27 F and 1492R which targeted 16 S rRNA gene fragments. Each PCR reaction consisted of 0.5 µL of 10 nM primer concentration, 12.5 µL of DreamTaq Green PCR master mix (Thermo Fisher Scientific), and 1 µL of Bovine Serum Albumin (BSA), and sterile double distilled water was added to achieve a final volume of 25 µL. Thermal cycling model Applied Biosystem Veriti 96-Well involved initial denaturation at 94 ℃ for 2 min, followed by 30 cycles of denaturation at 94 ℃ for 30 s, annealing at 44 ℃ for 30 s, and extension at 68 ℃ for 1 min 30 s. A final extension step was performed at 68 ℃ for 5 min. After amplification, the reaction products were stored at 4 ℃ and utilized within 24 h. Gel electrophoresis was conducted using a 1.5% agarose gel containing FluoroSafe DNA stain. Known DNA ladder fragments of 10,000 base pairs were run alongside the samples for size determination. Electrophoresis was carried out in Tris-borate-EDTA buffer at 100 V for 30 min and visualized under a gel documentation system. Successful PCR was confirmed by the presence of bands at approximately 1500 bp. The PCR products were sequenced on a Sanger Sequencing platform (Applied Biosystems, Apical Scientific, Malaysia).

To find the closest related strains, the generated 16 S rRNA sequences were BLASTed against the NCBI GenBank’s non-redundant nucleotide database. The isolate closest taxon was identified using identification method offer by EzBioCloud database^24^.

*Bacillus subtilis* NCIB 3610 and *Bacillus velezensis* CR-502 were used as the out group in the reconstruction of the phylogenetic tree, and the data of the highest relative strains were obtained for this purpose. After choosing the best phylogenetic model that fits this dataset, the alignment was completed using MEGA-X’s MUSCLE program. The phylogenetic tree was then reconstructed using MEGA-X’s maximum likelihood method between the isolate sequence and the nearest relatives^25^.

### Auto-aggregation and co-aggregation assay

To assess the potential of the new isolate to compete with pathogens for adhesion sites and establish a stable population within the fish gut. Auto-aggregation and co-aggregation assays were conducted using techniques outlined by Sam-on et al., with some modification^26^. For auto-aggregation, the bacteria were cultured in tryptic soy broth (TSB) at 37℃ for 24 h. Subsequently, the bacterial cells were collected through centrifugation at 5000×g for 10 min. The supernatant was discarded, and the cell pellets were washed twice with phosphate-buffered saline (PBS). The washed pellets were then suspended in the same buffer, and the absorbance (A_0_) value was measured at optical density (OD) 600 nm. A (10 mL) portion of the cell suspension was vortexed for 10 s and incubated at 37 ℃ for 2, 4, 6,8 and 24 h. After the incubation period, 3 mL of the upper layer of the suspension was transferred to another tube, and the absorbance (A_1_) was measured at OD 600 nm. The auto-aggregation was calculated using the following equation:

$$\mathrm{Auto}-\mathrm{aggregation}\:\left(\%\right)=\:1-\:({\mathrm A}_1/{\mathrm A}_0)\times100$$

Where A_0_ = Absorbance at 0 time, and A_1_ = Absorbance at 2, 4, 6, 8 and 24 h (OD 600 nm).

For the co-aggregation assay, the absorbance of the bacterial isolate (A_isolate_) and the suspension of the pathogenic bacterium (A_pathogen_) (*Streptococcus* sp. and *Aeromonas* sp.) was individually measured at OD 600 nm. Subsequently, an equal volume of the isolated bacterium and the pathogen were mixed in a conical tube for 10 s, followed by incubation at 37 ℃ for 2, 4, 6, 8 and 24 h. After the incubation period, 3 mL of the upper layer of the suspension was transferred to another tube, and the absorbance was measured at OD 600 nm (A_mix_). The co-aggregation percentage was calculated as outlined below:

$$\mathrm{Co}-\mathrm{aggregation}\;\left(\%\right)=1-\langle\:\left({\mathrm A}_{\mathrm{mix}}\right)/\left(\frac{{\mathrm A}_{\mathrm{isolate}}+{\mathrm A}_{\mathrm{pathogen}}}2\right)\rangle\:\times\:100$$

Where, A_mix_ = Absorbance after incubation. A_isolate_ = Absorbance of the tested isolate before mixing, A_pathogen_ = Absorbance of pathogen before mixing.

### Cell surface hydrophobicity assay

Cell surface hydrophobicity assay were conducted using techniques outlined in the methods described by Sam-on et al.,^26^. The bacterial isolates, cultured overnight, were collected by centrifugation at 5000×g for 10 min, washed twice with PBS, and then suspended in 10 mL Ringer’s solution (NaCl 8.6 g/L, KCl 0.3 g/L and CaCl_2_ 0.33 g/L). The absorbance was initially measured at OD 600 nm (A_0_). Subsequently, an equal volume of the solvent chloroform and the bacterial suspension were combined in a conical tube. The solution was thoroughly mixed by vortexing for 2 min and incubated at 37 ℃ for 24 h. Following incubation, the aqueous phase was separated, and the absorbance was measured at OD 600 nm (A_1_). The percentage of bacterial adhesion to the solvent was calculated using the formula below:

$$\mathrm{Hydrophobicity}\;\left(\%\right)=(1-\left(\frac{A_1}{A_0}\right))\times\:100$$

Where A_0_ = Absorbance at 0 time and, A_1_ = Absorbance at 24 h (OD 600 nm).

### Safety assessment

For safety assessment of the usage of new proposed probiotics strains in aquaculture, many traits should be assessed which include the antibiotic resistance traits and the ability to cause disease. Antibiotics resistance, ability in vitro to lysis red blood cells, and in vivo potential to cause disease were evaluated.

#### Antibiotic susceptibility test

The strains that should be proposed for use as probiotics should not have any antibiotic resistance trait. To assess the sensitivity of selected microbe for antibiotic sensitivity the disc diffusion technique was used. The susceptibility of the strain to antibiotics was assessed following the antimicrobial susceptibility testing standards set by the Clinical and Laboratory Standard Institute^27^, The antibiotics used in the susceptibility test were Cefoperazone (75 µg), Cefaclor (30 µg), Clarithromycin (15 µg), Imipenem (10 µg), Vancomycin (30 µg), Cefdinir (5 µg), Ofloxacin (5 µg), Pefloxacin (5 µg), Chloramphenicol (30 µg), Nitrofurantoin (300 µg), Moxifloxacin (5 µg), Tobramycin (10 µg), Nalidixic acid (30 µg), Tetracycline (30 µg), Streptomycin (10 µg), Gentamicin (10 µg), Clindamycin (2 µg), Lincomycin (15 µg), and Penicillin (10 µg). The inhibition zone was measured using a ruler and the result was converted to sensitive (S), intermediate (I) and resistance (R) matrix.

#### Pathogenic potential and safety

To assess the potential of the promising microbe to cause disease in target animal, two approaches were used, the blood hemolysis test and challenge through intraperitoneal injection. A hemolytic assay was conducted by streaking it onto agar plates supplemented with 7% sheep blood. These plates were then incubated at 37 ℃ for 48 h, and the presence of hemolytic zones were observed. The isolates were subsequently categorized based on α, β, or γ-hemolysis. Isolates exhibiting a green zone surrounding the colony were classified as α-hemolysis, while those with a clear zone were identified as β-hemolysis. Isolates that did not produce any zone around the colony were categorized as γ-hemolysis^19^.

To evaluate potential harmful effects of probiotic bacteria on fish, two groups of Nile tilapia fish (*Oreochromis niloticus*) were prepared. Each group contained 10 fish with an average body weight of 100 g. One group received an intraperitoneal injection of 0.1 mL of the bacterial strain (at a concentration of 10^8^ CFU/mL), while the other group of fish received injections of sterile PBS (pH 7.2) in the same volume as a control. Fish were observed daily for any clinical signs, and the mortality rate was recorded over a period of 10 days^19^.

### Statistical analysis

All the experiments were conducted in triplicates and repeated 2 times. All statistical analysis was conducted using SPSS. The results are shown as means ± SEM. One-way ANOVA was employed to compare different treatments, followed by Tukey’s multiple comparison test where applicable. Statistical significance was defined as *p* < 0.05.

### Ethical approval

The experimental procedures for fish in this study adhered to Egyptian legislation on ethics in fish use and handling and were approved by the Committee of Aquatic Animal Care and Use in Research at the Faculty of Aquatic and Fisheries Sciences, Kafrelsheikh University, Egypt (approval number: IAACUC-KSU-02224-2022). The study is reported in accordance with ARRIVE guidelines for animal research and all experiments were performed in accordance with relevant guidelines and regulations.

## Results

### Bacterial isolates

From examined samples, 22 different phenotypes were recovered. All the isolates were purified using streaking plate method and categorized based on Gram staining and endospore formation. All the isolates were kept in glycerol broth for further characterization.

### Extracellular enzymes

**Figure 1:**
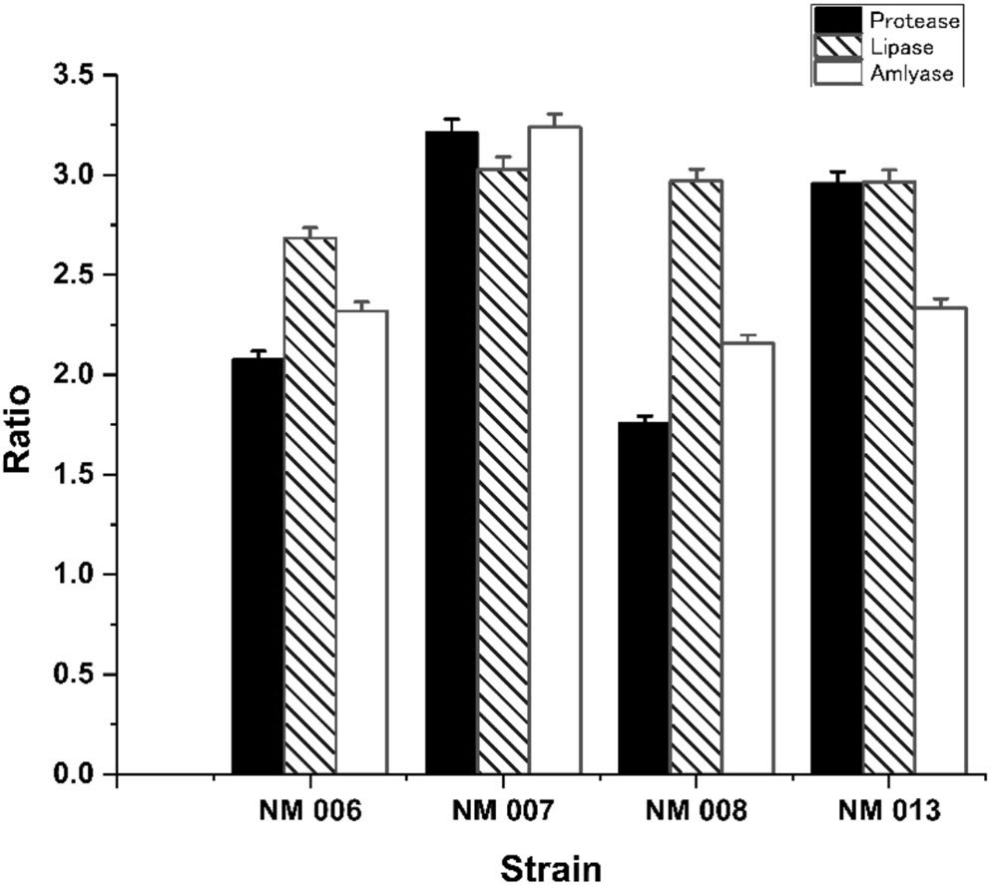
Ratio on enzymes activity of the isolates, calculated by dividing the substrate hydrolysis zone diameter by the colony diameter for each isolate and respective enzyme at 37 ℃ under aerobic stagnant condition. The columns (mean ± SEM, n  = 6).

Not all strains could produce the three important hydrolytic enzymes. Only four isolates are shown to have the ability to produce amylase, protease and lipase enzymes. From a closer look to Fig. 1, the strain coded NM007 showed the highest activity of all extracellular enzymes. The result expressed as the ratio of the clear zone diameter to the colony diameter for all tested enzymes and strains. The strain produced the highest ratio were selected for farther characterization.

### Phenotypic characterization

**Figure 2:**
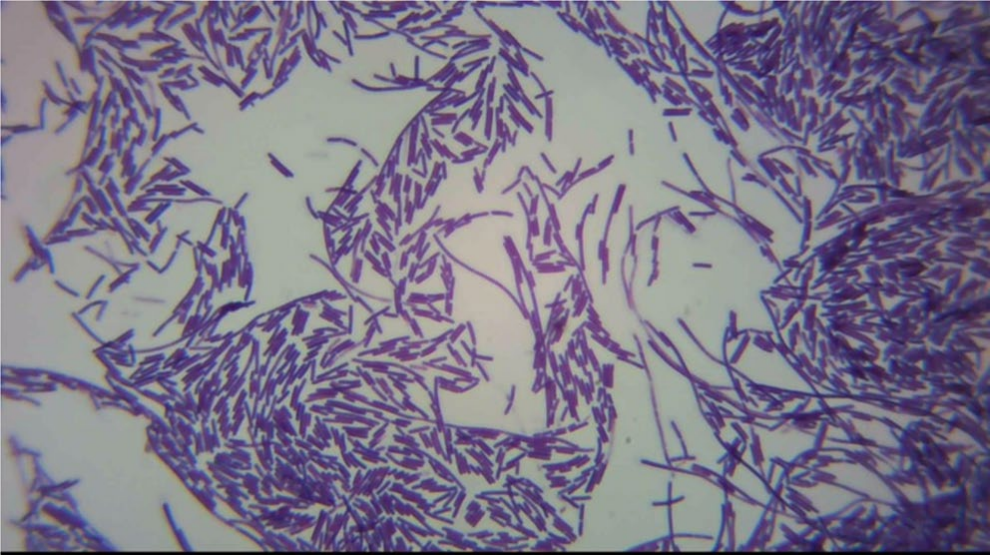
Oil immersion (100X) magnification of strain NM007 Gram-stained slide.

The strain NM007 was characterized phenotypically as a Gram-positive (Fig. 2), spore-forming rod morphology. It tested positive for oxidase, gelatin liquefaction, and the VP test. Detailed results of the biochemical tests are shown in Table 1.

**Table 1:** Biochemical and morphological characterization of bacterial isolates

Characteristics	Results	Characteristics	Results
Gram stain	+	Growth at 1% NaCl	+
Endospore stain	+	Growth at 2% NaCl	+
Catalase	-	Growth at 3% NaCl	+
Oxidase	+	Growth at 4% NaCl	+
Gelatin	+	Growth at 6% NaCl	+
O-F test	-	Growth at 1% Bile salts	+
Protease	+	Growth at 2% Bile salts	+
Lipase	+	Growth at 3% Bile salts	+
Amylase	+	Growth at 4% Bile salts	+
Tannase	+	Growth at pH 6	+
Voges–Proskauer	+	Growth at pH 7	+
Methyl red	-	Growth at pH 8	+
Motility test	+		

Positive (+) and negative (-)

### Molecular characterization

**Figure 3:**
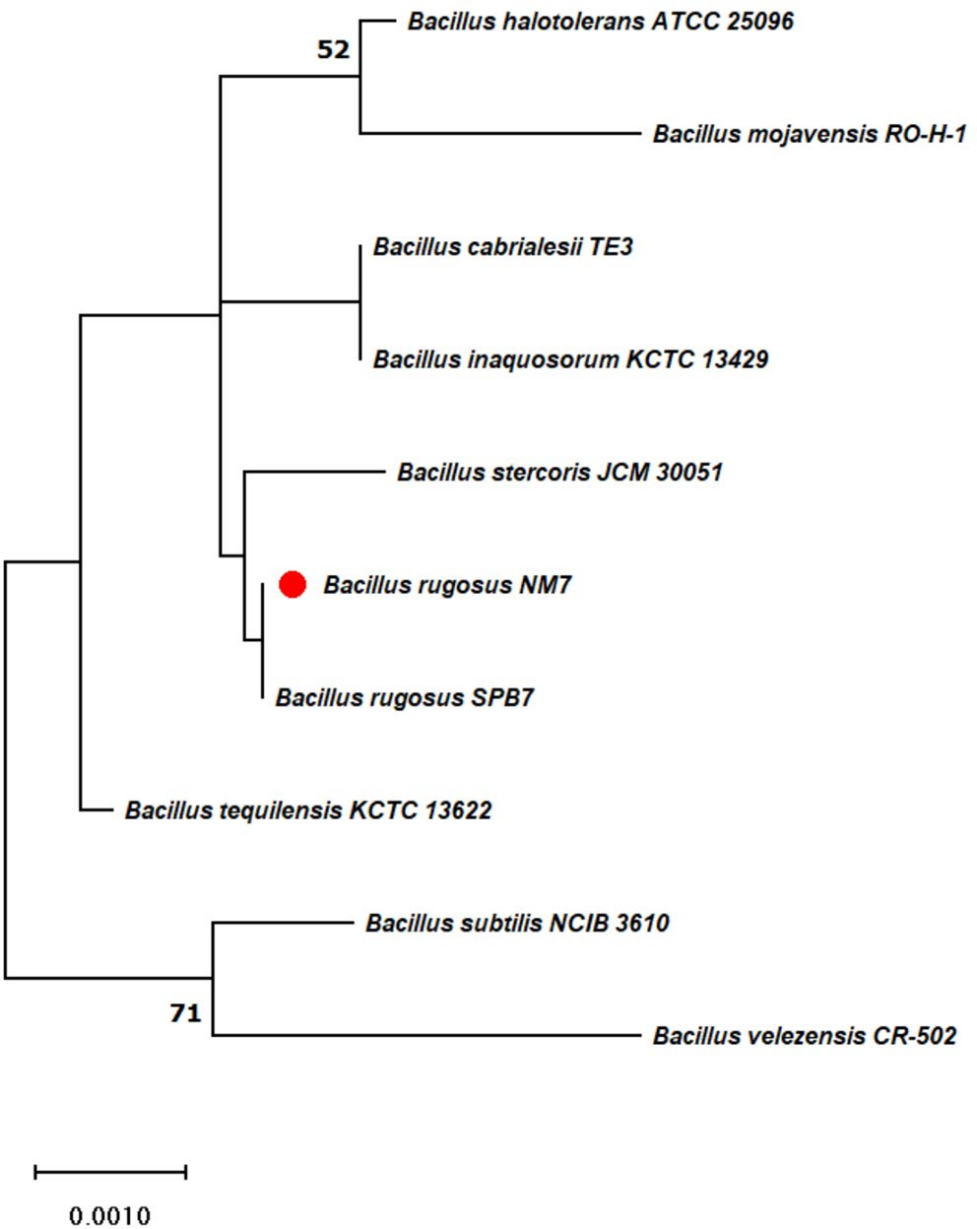
Phylogenetic relationship between *Bacillus rugosus* NM007 and the closely related taxa, the tree was drawn to scales using Hasegawa-Kishino-Yano model and using Maximum Likelihood method. All positions with less than 95% site coverage were eliminated using MEGA-X.

The identification of the isolate NM007 using EzBioCloud search revealed that the closest neighbor to this isolate is *Bacillus rugosus* SPB7 with similarity 99.82%. The phylogenetic relationship between the isolate and the closely related species among genus *Bacillus* is shown in Fig. 3. The sequence was submitted to GenBank, and the strain accession number is LC18114.

### Co-aggregation assay

**Figure 4:**
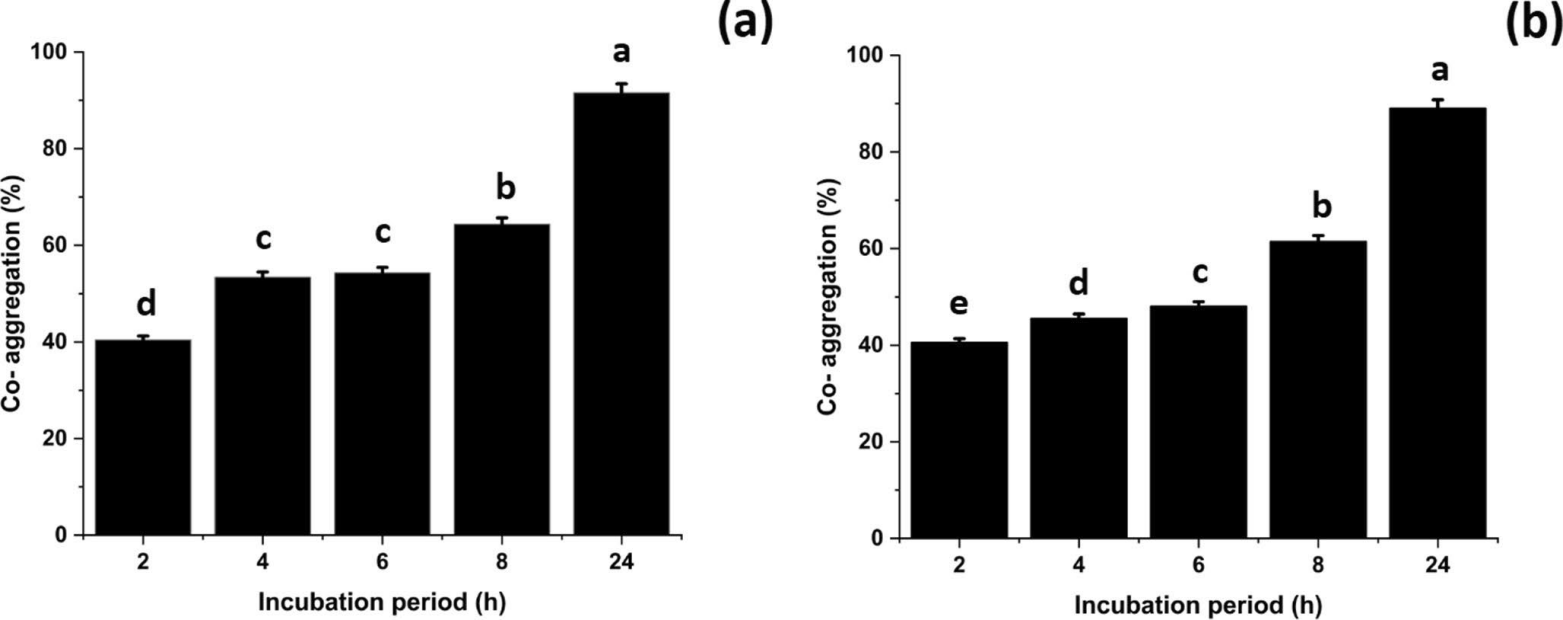
Co-aggregation reaction pattern change of *B. rugosus* NM007 over time (24 h incubation period) in presence of the fish pathogens Streptococcus (**a**) and Aeromonas (**b**) strains at 37 ℃ under aerobic stagnant condition. The columns (mean ± SEM, n  = 6) with different letters are significantly different (p  < 0.001, one-way ANOVA).

The co-aggregation rate of *B. rugosus* NM007 strain with two fish pathogens namely, *Aeromonas* sp. and *Streptococcus* sp., was studied. The co-aggregation started to be observed from 2 h with values of 40.5% and 40.4% respectively and continued to increase to reach the maximum after 24 h incubation with values 89.03% and 91.6%, respectively. (Fig. 4).

### Autoaggregation and cell surface hydrophobicity

**Figure 5:**
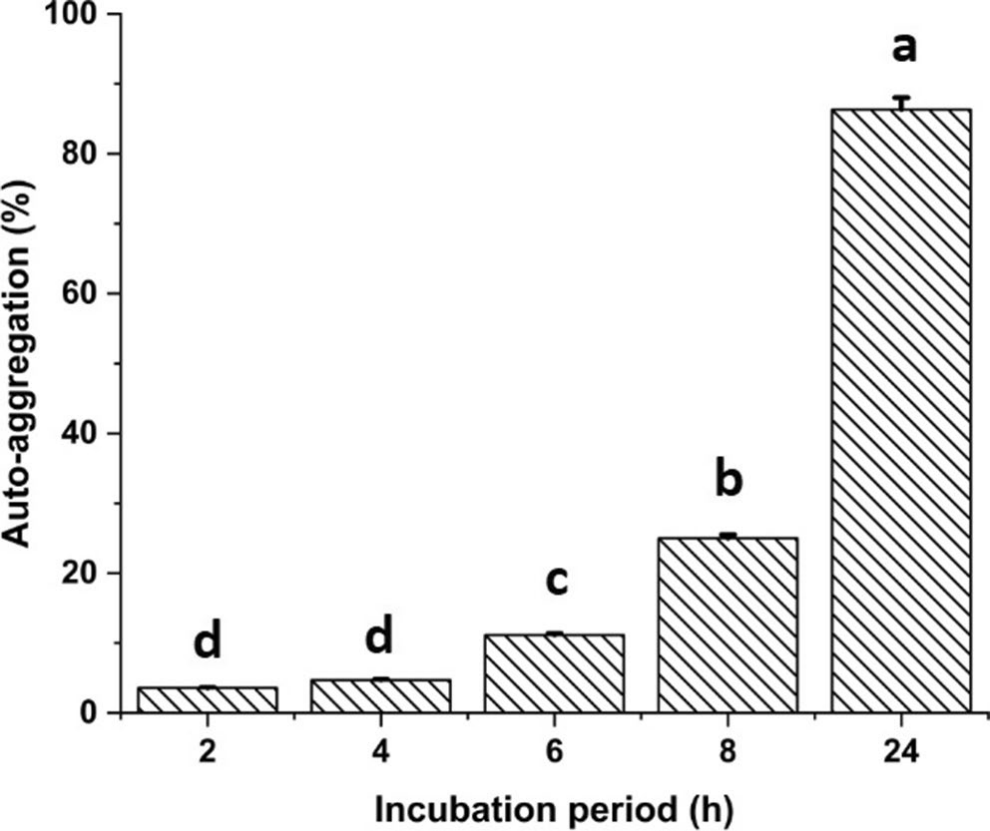
Auto-aggregation pattern of the *B. rugosus* NM007 strain over time (24 h incubation period) at 37 ℃ under aerobic and stagnant condition. The columns (mean ± SEM) with different letters are significantly different ( p  < 0.001, one-way ANOVA) The columns (mean ± SEM, n  = 6).

The auto-aggregation rate of *B. rugosus* NM007 was studied. Auto-aggregation started to be observed from 2 h with value of 3.6% and continued to increase to reach the maximum after 24 h incubation with value 86.5% (Fig. 5). The NM007 strain showed cell surface hydrophobicity also was 89.8% after 24 h.

### Safety assessment of new probiotics

#### Antibiotic susceptibility test

Table 2 list the number of the antibiotics used on antibiotics assay. From the results, *B. rugosus* NM007 does not show antibiotic resistance pattern with sensitivity to sixteen antibiotics, and intermediate resistance to two antibiotics namely Clindamycin and Lincomycin. It showed only resistance to penicillin.

**Table 2:** Antibiotic susceptibility test results

**No#**	**Antibiotic**	**Full name**	**Result**	**Clear zone diameter (mm)**
1	CPZ75	Cefoperazone	S	29
2	CF30	Cefaclor	S	35
3	CLR15	Clarithromycin	S	28
4	IMP10	Imipenem	S	35
5	VA30	Vancomycin	S	26
6	CDR5	Cefdinir	S	28
7	OF5	Ofloxacin	S	30
8	PF5	Pefloxacin	S	30
9	C30	Chloramphenicol	S	28
10	F300	Nitrofurantoin	S	26
11	MXF5	Moxifloxacin	S	32
12	TOB10	Tobramycin	S	28
13	NA30	Nalidixic acid	S	27
14	TE30	Tetracycline	S	26
15	S10	Streptomycin	S	28
16	CN10	Gentamicin	S	22
17	DA2	Clindamycin	I	16
18	L15	Lincomycin	I	16
19	P10	Penicillin	R	10

*S* Susceptible, *I *Intermediate, and *R* Resistant

#### Pathogenicity

**Figure 6:**
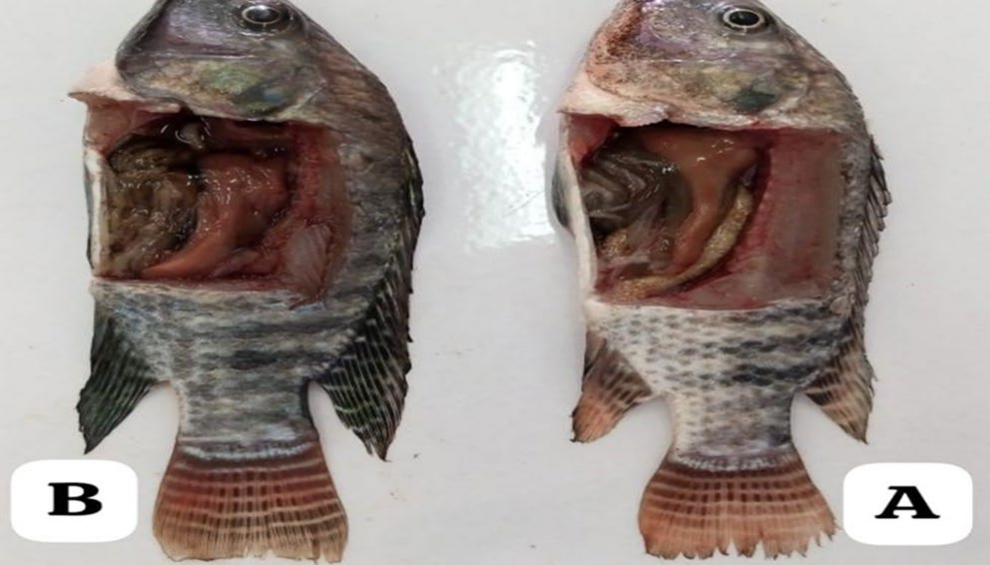
Pathogenicity test: No clinical signs on fish or in internal organs, **A**: Control fish that injected with PBS, **B**: injected fish with bacterial strain *B. rugosus*.

No clinical signs (such as swelling, bleeding, wounds, or loss of scales and mucus) were detected in either the experimental or control fish during the *in vivo* biosafety assessment. Additionally, there were no mortalities recorded. This indicates that the isolate was non-pathogenic (Figure 6). However, hemolytic activity results in the strain showed α-hemolytic activity.

## Discussion

Approximately 17% of the animal protein required by humans is provided by aquaculture, however various stresses, including disease and a scarcity of larvae, impede its growth^28^. Historically, the aquaculture sector relied heavily on the use of antibiotics to limit disease spread and protect fish fries and larvae in their early stages. However, due to increasing concerns about antibiotic and antimicrobial resistance (AMR), scientists are now looking for safer and more effective strategies to manage infections and boost fish development^29,30^.

To achieve this purpose, probiotic technology began to evolve, either to fight pathogens or to promote fish health and optimize the benefit from resources^31^. For this reason, the current study was done to identify new probiotic strains that can assist maximize the benefit of feed and reduce waste during fish development while also minimizing the environmental impact of aquaculture.

The strains that exhibited synthesis of all necessary enzymes were Gram-positive, spore-forming, bacteria and had a phenotypic character of *Bacillus* spp. which is known and been manufactured commercially and utilized successfully in aquaculture applications^32,33^. The results of phenotypic characterization revealed that the strain belongs to genus *Bacillus*, which was validated using molecular identification. The identification classified this isolate among the *Bacillus rugosus* species that has never been tested or utilized for aquaculture application. That species was firstly mentioned by Bhattacharya et. Al., as a novel species. One of the traits that makes this species suitable for use in aquaculture is its capacity to synthesis the three primary hydrolytic enzymes^34^. Specifically, lipase, amylase, and protease, which are essential for the full utilization of feed ingredients. The introduction and augmentation of these valuable enzymes will have a beneficial effects on the digestive system and feed utilization^35^. This could be achieved through the usage of live microorganisms in the diet to act as source of enzymes and considered as a golden key for many aquaculture problems related to fish digestion and feed consumption.

Other essential criteria for probiotics include their capacity to survive in the stomach and the gut bile salt content^36^. The results showed that *B. rugosus* NM 007 can grow well in a range of pH that covers slightly acidic or alkaline pHs. This ability to survive in acidic conditions enables the probiotic organism to persist in the host’s upper gastrointestinal tract^37,38^. Furthermore, salt tolerance is vital for various kinds of applications, such as fresh or marine aquaculture. *B. rugosus* NM007 characterization has demonstrated its ability to grow in salt levels of up to 60 ppt, making it an excellent choice for freshwater and marine farms. Regarding the survival inside the gut, good probiotics should show tolerance to a range of bile salt concentrations. More bile salts inhibit the growth of bacteria as well as affecting bacterial membranes, proteins and induces oxidative stress^39,40^. So, for a probiotic to live and grow in the gut, it should handle at least 0.3% bile^38,41^. The strain *B. rugosus* NM007 exhibited bile tolerance up to 4%, suggesting its capability to survive and thrive in the host animal’s gut.

Cell surface hydrophobicity is another important characteristic which also plays a significant role in bacteria ability to adhere to surfaces successfully. These characteristics might help the bacteria live in the stomach and intestines, allowing them to benefit the host’s health^42^. In the current study, *B. rugosus* NM007’s total cell surface hydrophobicity was found to be 89.8%, indicating that it has the potential to cling to the intestinal lining and attach itself. In other words, the more hydrophobicity cell surface, the higher the probability of localizing the intestine. This result is supported from studies which showed that certain *Bacillus* strains used as probiotics exhibited high hydrophobicity percentages over 50%^43,44^.

To adhere to intestinal epithelial cells, auto-aggregation properties are very important for successful probiotics. In addition, to their capacity to co-aggregate with the pathogens may create a barrier that prevent pathogenic microorganisms from invading the digestive tract^45,46^. In the current study *B. rugosus* NM007 strain showed a high percentage for auto-aggregation test with 86.5% after 24 hrs as well as a high co-aggregation with both *Aeromonas* sp. and *Streptococcu*s sp. with 89.03 % and 91.6 % respectively. These findings supported a previous study that showed a high percentage of auto-aggregation and co-aggregation of some *Bacillus* species with *Aeromonas* after a 24 hrs incubation period^36^.

To ensure the bacterial isolate is safe for aquaculture, safety assessment tests, such as antibiotic sensitivity and hemolytic tests, were performed. Some bacteria found in commercial probiotic products have been identified as bacterial species with antibiotic resistance and virulence characteristics^47^. Although some research has demonstrated that antibiotic-resistant bacteria can pass resistance genes to other bacteria, leading to the development of antibiotic resistance^48^. To ensure safety, a probiotic should not exhibit this characteristic. In this study *B. rugosus* NM007 was susceptible to over sixteen different antibiotics which minimize the risk of transferring the antibiotic resistance genes to either fish or human pathogens^49,50^. For the hemolysis assessment, it is essential to show a weak or a lack of hemolytic activity to confirm that the strains are non-virulent and do not produce hemolysin, a toxin harmful to cells^51^. *B. rugosus* NM007 showed a weak α-hemolysis, this characteristic is generally associated with a lower pathogenic potential compared to beta hemolytic bacteria, which fully lyse red blood cells and may pose higher risks. Despite this, certain beta hemolytic strains like *Bacillus cereus* and *Bacillus subtilis* are utilized in aquaculture without causing any clinical signs^52–54^. Their successful application in aquaculture suggests that the hemolysis pattern alone does not necessarily equate to harmful effects. Additionally, the strain’s safety can be further validated by demonstrating its lack of pathogenicity through the safety tests, such as assessing its impact on fish health in controlled environments and ensuring it does not exhibit any harmful effects upon injection. Including these details will provide a comprehensive rationale for the probiotic’s safety and efficacy, addressing potential concerns and reinforcing the validity of the chosen strain, and the strain *B.rugosus* NM007 it is showed no clinical signs or mortalities when injected in fish after 10 days monitoring trial.

## Conclusion

*Bacillus rugosus* NM007, showed promising probiotic characteristics. It passed safety assessments and demonstrated tolerance to various physiological conditions. *B. rugosus* NM007 could endure 4% bile salt concentration and salinity up to 60 ppt. It also displayed substantial abilities in auto-aggregation and cell surface hydrophobicity. It showed susceptibility to all common antibiotics and had proteolytic amylolytic and lipolytic activity. Molecular analysis provided valuable insights into the evolutionary relationship of *B. rugosus*. Based on these findings, *B. rugosus* stands as a new promising probiotic candidate for aquaculture practices.

## Data Availability

The data presented in this study are available on request from the corresponding author.
